# Outcomes and validity of risk stratification tools for endoscopic submucosal dissection of early gastric cancer in Western Australia

**DOI:** 10.1002/jgh3.70034

**Published:** 2024-11-15

**Authors:** Ciaran Judge, Abir Halder, Puraskar Pateria, Tzeng Khor, Niroshan Muwanwella, Marcus Chin, Krish Ragunath

**Affiliations:** ^1^ Department of Gastroenterology Royal Perth Hospital Perth Western Australia Australia; ^2^ Department of Anatomical Pathology Royal Perth Hospital Perth Western Australia Australia; ^3^ Faculty of Health Sciences, Curtin Medical School Curtin University Perth Western Australia Australia

**Keywords:** endoscopic submucosal dissection, ESD, gastric cancer, stomach

## Abstract

**Background and Aim:**

Endoscopic submucosal dissection (ESD) has become the treatment of choice for many superficial gastric neoplasms. Clinical outcomes are increasingly comparable between Japanese and Western series; however, data are lacking on the validity of risk stratification tools in Western cohorts. We aimed to evaluate clinical outcomes, explore risk stratification, and compare our data with published Western series.

**Methods:**

We conducted a retrospective, observational cohort study in a single tertiary referral center over a 13‐year period. Primary outcomes were rates of en bloc, complete (R0) and curative resection. Secondary outcomes included adverse events, recurrence, metachronous lesions, eCura grades, and ESGE criteria. A comparative analysis was performed with existing published series from Western centers.

**Results:**

Totally 112 patients were included in the study cohort. 50.9% were male, 87.5% Caucasian, and median age was 75.5 years (IQR 14.3 years). Lesions were predominantly antral (36.6%) or body (35.7%); median size 20 mm (IQR 15 mm). Rates of en bloc, R0 resection, and curative resection were 96.4%, 89.3%, and 78.6% (identical between eCura and ESGE), respectively. Adverse events occurred in 5.8%, recurrence in 0%, and metachronous lesions in 9.9%. Our data compared favorably with a review existing Western series, which illustrates increasing adoption of ESD and stable outcomes over time.

**Conclusion:**

ESD represents a safe and effective method of treatment for gastric neoplasia in the Western setting. This study highlights the potential for excellent outcomes in a single center with a heterogeneous patient cohort and supports the use of eCura in guiding post procedural management.

## Graphical abstract

We present the outcomes for endoscopic submucosal dissection (ESD) for early gastric cancer (EGC) in a single tertiary referral centre, with positive chronological comparison made to international Western series.

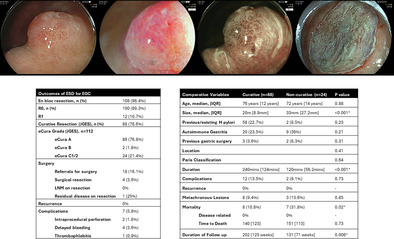

## Introduction

Since its inception in Japan in the 1990s, endoscopic submucosal dissection (ESD) has become the treatment of choice for gastric neoplasia with low risk of lymph node metastasis (LNM).^1^, ^2^, ^3^ Early gastric cancer (EGC) is defined as carcinoma invading no deeper than the gastric submucosa, regardless of lymph node status.^4^ ESD for EGC results in superior technical and clinical outcomes when compared with endoscopic mucosal resection (EMR) and gastrectomy, in addition to fewer adverse events and enhanced health‐related quality of life.^3^, ^5^, ^6^

After initial concerns over safety and efficacy, multiple series have demonstrated that outcomes from Western centres have become generally comparable with those from East Asia.^3^, ^7^, ^8^, ^9^, ^10^ However, differences in demographics, natural history and healthcare utilization raise the possibility that Japanese data may not be entirely applicable to a Western population.

Prior to resection, image enhanced endoscopy (IEE) has been shown to be accurate in predicting lesion characteristics, including depth of invasion. Yet, approximately 20% of ESD result in non‐curative resections.^3^, ^11^ This can occur despite complete or R0 removal of a lesion due to the histopathological presence of features associated with higher risk of LNM, for example, poor differentiation, lymphovascular invasion, or ulceration. To guide further management in these cases, scoring systems such as eCura have been developed to define and predict ‘curability’, that is, risk of LNM and recurrence.^12^ This system was validated in a Japanese population, and so data on its utility in Western centers remain limited. Similar risk stratification tools have been developed by the European Society of Gastrointestinal Endoscopy (ESGE), and efforts are underway to explore dedicated tools for Western populations.^3^, ^13^

To support an evidence‐based expansion of ESD for EGC, real‐world data on safety and efficacy from Western centers are essential. This study aimed to describe outcomes of gastric ESD in a multicultural, tertiary referral center in Australia and to explore the utility of risk stratification tools in this cohort.

## Methods

We conducted a retrospective, single‐center, observational cohort study of patients who underwent ESD for EGC from January 2010 to September 2023. This study took place in a single tertiary referral center in Perth, Australia. Demographics and relevant clinical data were collected and analyzed from patient records. These included Helicobacter Pylori (H pylori) status (as defined presence of H pylori on histopathological analysis during diagnostic workup or on ESD specimen), previous gastric surgery history, prior ESD and EMR history, and history of autoimmune gastritis. Endoscopic, pathological, and procedural characteristics were also recorded. Histopathology was defined according to the World Health Organization (WHO) classification.^14^

### Primary and secondary outcomes

The primary outcomes for this study were endoscopic *en* bloc, R0, and curative resection rates in all ESDs performed. En bloc resections were those where the lesion was endoscopically judged to be fully removed in a single piece. R0 or complete resection was where lateral and deep margins were clear of neoplasm. Curative lesions were those described by eCura A or B criteria, with all other lesions categorized as eCura C1 or 2.

Secondary outcomes included procedural complications, post ESD surgery, local recurrence, and metachronous lesions. Local recurrence was defined as the presence of endoscopic or histological evidence of gastric neoplasm at the site of index ESD during follow‐up endoscopy. Metachronous lesion was defined as any new neoplasm in an area other than the site of index ESD during follow‐up endoscopy. Recorded complications included perforation during ESD, acute bleeding (within the first 24 h), and delayed bleeding (greater than 1 week following ESD). Other events such as follow‐up times, death from gastric cancer, and other causes were also described.

### Inclusion and exclusion criteria

**Figure 1:**
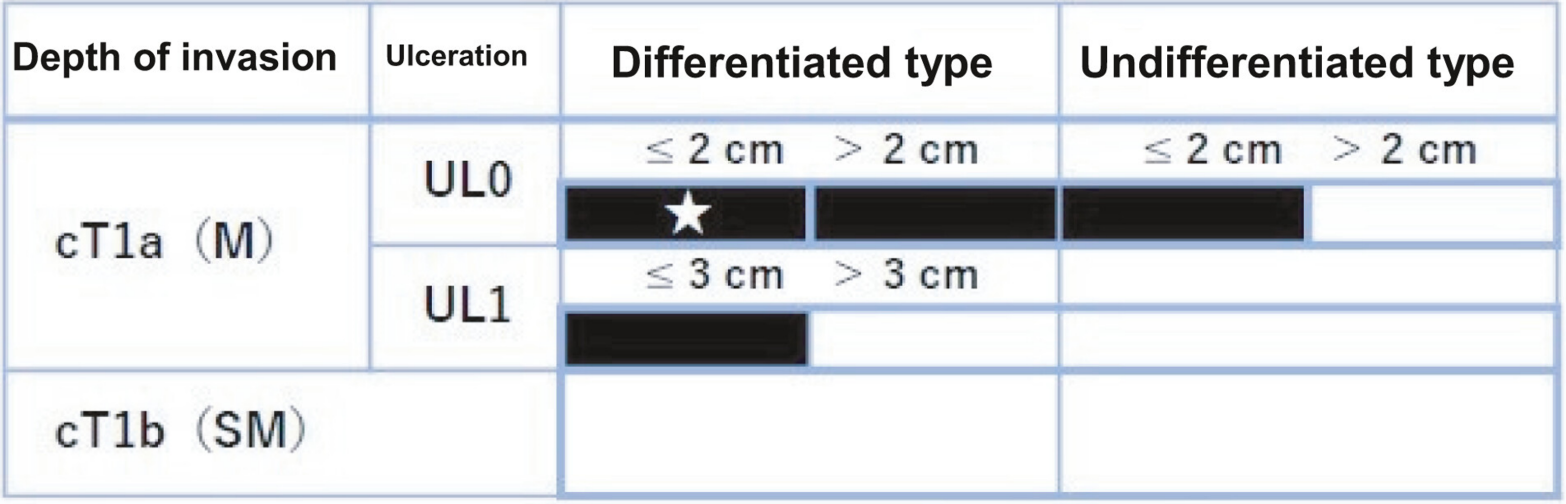
Absolute Indications and Expanded Indications for Treating EGC with ESD. Figure depicting indications and expanded indications for ESD of early gastric cancer as per Japanese Gastroenterological Endoscopy Society (JGES) guidelines. cT1a, 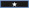 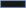, cT1b, 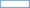.

Cases were included if ESD was performed for lesions that were diagnosed endoscopically and histologically as EGC. EGC was defined according to the Japanese Gastroenterological Endoscopy Society (JGES) definition of carcinoma invading no deeper than the submucosa, regardless of lymph node status.^4^ Low‐grade (LGD) or high‐grade dysplasia (HGD) corresponded to Tis. Expanded criteria for resection were defined by the JGES guidelines 2016^1^ (Fig. 1). ESD for gastric lesions that did not meet these histopathological definitions were not included in this study. Lesions were described according to location, size, and Paris classification.

### ESD procedure

ESD was performed by two operators: MC (2010–2023) and NM (2016–2023). Referrals for ESD underwent additional endoscopic evaluation to assess for ESD suitability as per the absolute and expanded criteria. ESD was performed using a Fujinon 360Z adult gastroscope (ELUXEO processor). DualKnife‐J (Olympus Medical) and IT knife (Olympus Medical) were used for resection. All lesions were marked 2–3 mm outside the demarcation line of the lesion prior to resection and lifted using indigo carmine and gelofusine. Dilute Adrenaline was used in the lifting solution at the periphery of the lesion prior to initial mucosal incision.

Various traction methods were used at the endoscopist's discretion including clip+snare, cli + suture line, etc. Patients were admitted to hospital post ESD and were started on either proton pump inhibitor (PPI) infusion or twice daily PPI. Patients were started on clear fluids on the day of the procedure and slowly upgraded to a normal diet over 3–4 days. If the patients were started on bd PPI, they were discharged home the following day, and if the patients were on PPI infusion, they were admitted to hospital for 72 h.

### Histopathological assessment and staging

**Figure 2:**
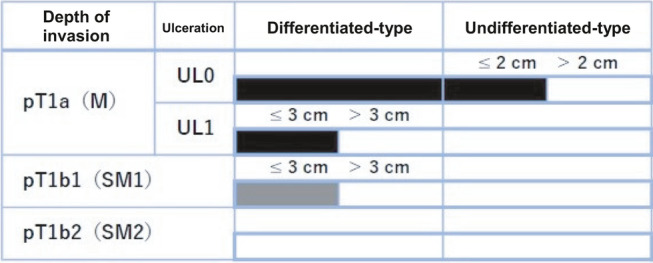
eCura criteria for curability. Figure depicting eCura grade according to Japanese Gastroenterological Endoscopy Society (JGES) Guidelines 2020. Lesions are confined to en bloc resection and HM0, VM0, Ly0, and V0. pT1a (M), intramucosal cancer (histopathological diagnosis); pT1b (SM), submucosally invasive cancer (histopathological diagnosis). UL, finding of ulceration (or ulcer scar); UL0, absence of ulceration or ulcer scar; UL1, presence of ulceration or ulcer scar (1). Note is made of misspelling of eCura as ‘eCure.’ eCureA*, 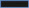; eCureB*, 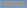; eCureC‐2, 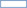.

Following ESD, all specimens were assessed by one of two pathologists with a special interest in GI pathology. Endoscopic curability of EGC was determined according to the eCura staging system (A, B, C1, C2) as described in the JGES guidelines 2020 (Fig. 2).^1^, ^12^ Lesions were considered curative if they were eCura A or B.

**Figure 3:**
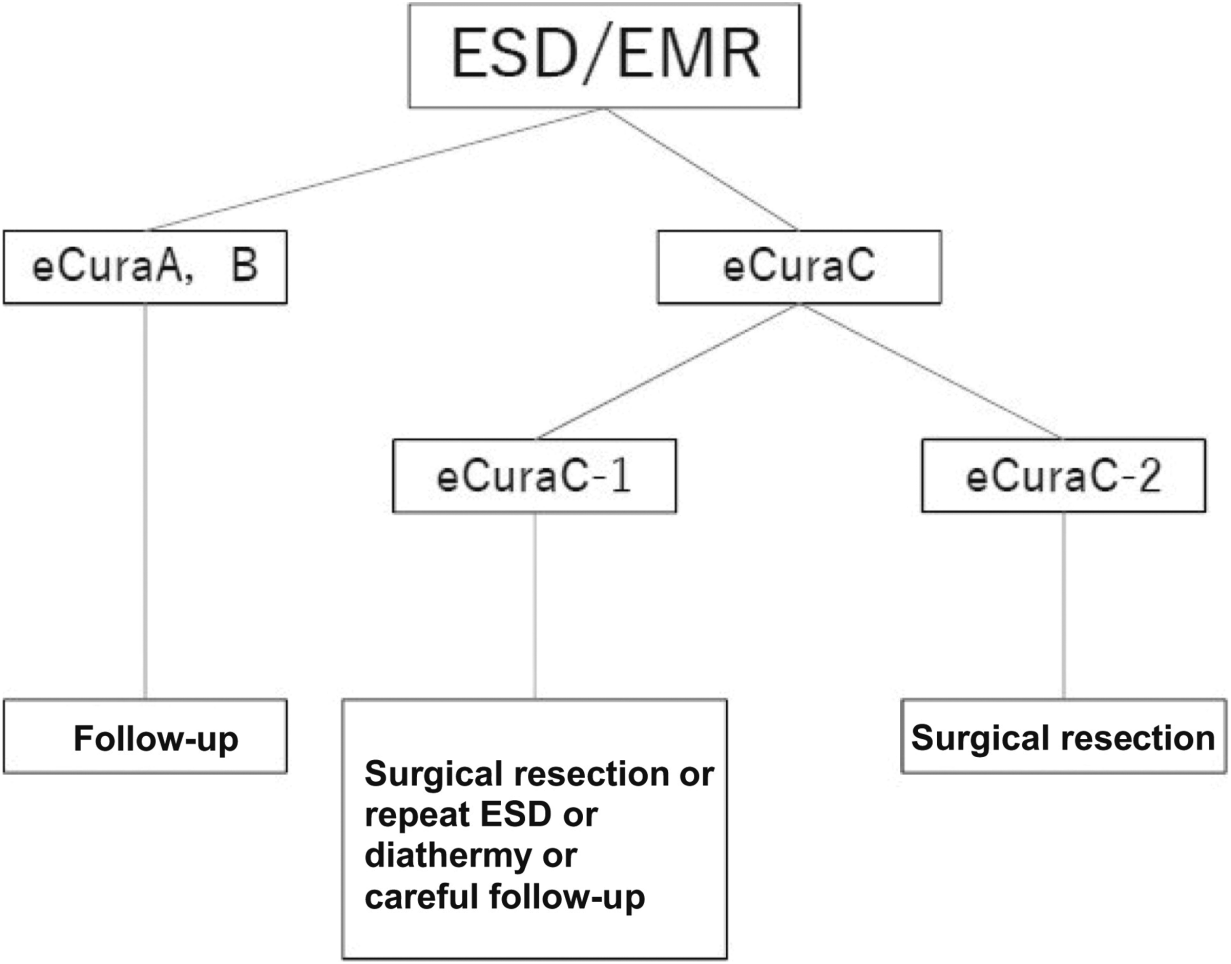
Japanese Gastroenterological Endoscopy Society (JGES) algorithm following ESD. JGES guidelines on management of lesions following ESD.

Lesions were considered eCura A if they were resected en bloc and met the following conditions according to JGES guidelines: (i) predominantly differentiated type, pT1a, UL0, HM0 VM0, Ly0, V0, regardless of size; (ii) long diameter ≤2 cm, predominantly undifferentiated type, pT1a, UL0, HM0, VM0, Ly0, V0; or (iii) long diameter ≤3 cm, predominantly differentiated type, pT1a, UL1, HM0, VM0, Ly0, V0.^1^ Lesions were considered eCura B if they were resected en bloc and were ≤3 cm in long diameter, predominantly of the differentiated type, and satisfy the following criteria: pT1b1 (SM1) (within <500 μm from the muscularis mucosae), HM0, VM0, Ly0, and V0. Lesions that did not meet the criteria for eCuraA or B were eCuraC. If lesions met criteria for eCuraA or B but were not resected en bloc or had positive horizontal margins, they were defined as eCuraC‐1. All other lesions were eCuraC‐2. Further management was based on JGES guidelines (Fig. 3).^1^

Patients with eCura grades A and B were followed up with an initial 3‐month gastroscopy followed by annual gastroscopy and computed tomography (CT) surveillance for recurrence and metastasis. Patients with an eCuraC‐1 and 2 scores were routinely referred for surgery in line with JGES guidelines (Fig. 3). However, a similar surveillance strategy was also offered in patients who were unfit for surgery or declined further treatment.

We also looked at other risk stratification tools to determine if there were differences in outcomes. The ESGE guidelines on ESD for superficial gastrointestinal lesions categorize patients into very low/low (curative), low and high (non‐curative) categories. W‐eCura is a modified version of the eCura system that has been described recently to apply to a non‐Western cohort. The most notable difference with eCura is that lesions demonstrating submucosal invasion are considered to be low risk until they display depth of invasion of >1000um. This scoring system has yet to be validated. We calculated ESGE and W‐eCura grades to determine if there were any significant differences between the scoring systems.

### Review and comparison of existing data

A literature review of published data was conducted using Pubmed and Embase using the search terms ‘gastric’ and ‘ESD’. Relevant systematic reviews (SR) were included and combined with subsequent published data from inception until December 2023. A systematic review and meta‐analysis by Zullo et al. was explored and granular data extracted which reported outcomes to October 2019.^15^ A review of subsequently published series was added from this date until December ‘23. Studies were eligible for inclusion if they were from Western centers examining outcomes of ESD for EGC and were full articles available in English, Italian, or Spanish; on humans only; with at least five patients. Articles comparing ESD with different types of resection or comparing different subtypes of ESD technique were excluded. Articles containing combined outcomes for ESD of epithelial and non‐epithelial lesions were assessed and outcomes for EGC was extracted where possible. Studies were then analyzed and compared over time periods (2008–2014, 2015–2019 and 2020‐December 2023). These were then compared with our series to assess differences in outcomes.

### Statistical analysis

For all outcomes assessed, absolute (n) and percentages (%) were calculated for categorical variables and mean ± SD, median and interquartile ranges were calculated for continuous variables. Chi‐square or Fisher's exact tests were used to assess differences in categorical variables, and Student's *t* test or Mann–Whitney tests were used for continuous variables. Regression analysis was used to determine relationships between variables. A significant *P* value was defined as <0.05.

## Results

### Patient and lesion characteristics

A total of 112 ESDs were attempted for EGC over a 13‐year period. 50.9% of patients were male, with a median age of 75.5 years [IQR 14.3] (Table 1). Most patients were Caucasian (87.5%) or East Asian (8.9%); 19.8% had an existing or previous diagnosis of Helicobacter pylori, and 26.4% had a history of autoimmune gastritis. The majority of lesions were in the antrum (36.6%) or body (35.7%) and were flat in morphology (Paris OIIa – 67.3%). The median size was 20 mm, ranging from 9 to 130 mm. Most lesions were adenocarcinoma (75.9%) and were well or moderately differentiated (86.7%), and most were confined to the mucosa (pT1a – 61.3%, or intraepithelial neoplasia – 24.3%).

**Table 1:** Demographics and lesions characteristics

Demographics + Lesion characteristics (*n* = 112)	
Age at procedure, median [IQR]	75.5 [14.3]
Gender, *n* (%)	
Male	57 (50.9%)
Female	55 (49.1%)
Ethnicity	
Aboriginal or Torres strait	1 (0.9%)
African	1 (0.9%)
Caucasian	98 (87.5%)
East Asian	10 (8.9%)
Indian	1 (0.9%)
Middle Eastern	1 (0.9%)
History of *Helicobacter pylori*, *n* (%)	19 (19.8%)
Autoimmune gastritis, *n* (%)	29 (26.4%)
Anticoagulation, *n* (%)	
Yes	18 (17%)
DOAC	13 (12%)
Warfarin	5 (5%)
No	88 (83%)
Antiplatelets, *n* (%)	
Yes	23 (22%)
Aspirin	18 (17%)
Other	2 (2%)
Dual antiplatelet	3 (3%)
No	82 (77%)
Previous gastric surgery, *n* (%)	4 (4%)
*Lesion characteristics*	
Lesion location, *n* (%)	
Antrum	41 (36.6%)
Body	40 (35.7%)
Incisura	16 (14.3%)
Cardia	13 (11.6%)
Fundus	2 (1.8%)
Polyp morphology (Paris), *n* (%)	
Is	15 (15.3%)
IIa	66 (67.3%)
IIb	12 (12.2%)
IIc	5 (5.1%)
Lesion size, mm, median [IQR]	20 mm [15 mm)
Histopathology, *n* (%)	
Low grade dysplasia	9 (8%)
High grade dysplasia	18 (16.1%)
Adenocarcinoma	85 (75.9%)
Tumor differentiation, *n* = 83	
Well/Moderate	72 (86.7%)
Poor	11 (13.3%)
Tumor stage, *n* = 111	
T1a	69 (62.2%)
T1b	14 (12.6%)
T2	1 (0.9%)
Intraepithelial Neoplasia	27 (24.1%)
Depth of Invasion, um, [IQR]	900um [1100um]
Duration, mins, median [IQR]	120 min [80 min]
Type of anesthetic	
General anesthetic	112 (100%)

Table describing characteristics of patients, lesions and procedures. Staging based on WHO TNM staging system.

### Procedural data

All resections were performed by one of two interventional endoscopists. All patients received a general anesthetic. Median duration was 120 min, ranging from 24 to 510 min. Three procedures were abandoned due to severe submucosal fibrosis preventing safe resection.

### Outcomes

Over the study period, rates of en bloc, R0, and curative resection (as per eCura) were 96.4%, 89.3%, and 77.7%, respectively (Table 2).

**Table 2:** Outcomes of lesions and patients post ESD

Outcomes	
En bloc resection, *n* (%)	108 (96.4%)
Resection, *n* (%)	
R0	100 (89.3%)
R1	12 (10.7%)
Curative resection (JGES), *n* (%)	88 (78.6%)
eCura grade (JGES), *n* = 112	
eCura A	86 (76.8%)
eCura B	2 (1.8%)
eCura C1	2 (1.8%)
eCura C2	22 (19.6%)
ESGE risk grade, *n* = 112	
Curative (low/very low risk)	88 (78.6%)
Local risk resection	2 (1.8%)
Non‐curative/high risk	22 (19.6%)
W‐eCura, *n* = 112	
A	86 (76.8%)
B	2 (1.8%)
C1	3 (2.7%)
C2	21 (18.8%)
Surgery	
Referrals for surgery	18 (16.1%)
Surgical resection	4 (3.6%)
LNM on resection	0%
Residual disease on resection	1/4 (25%)
New lesions, *n* (%)	11 (9.9%), *P* = 0.85
eCura A	8 (7.2%)
eCura B	0
eCura C1	0
eCura C2	3 (2.7%)
Recurrence, *n* = 96	0%
Complications	7 (5.8%)
Intraprocedural perforation	2 (1.8%)
Delayed bleeding	4 (3.6%)
Thrombophlebitis	1 (0.9%)
Duration of follow‐up, weeks [SD]	158 [119 weeks], *P* = 0.006
eCura A	201 [125]
eCura B	213 [191]
eCura C1	87 [2]
eCura C2	131 [71]

Table describing outcomes including resection status, curative status and classification according to eCura, W‐eCura, and ESGE systems.

Complications occurred in seven cases (5.8%), including intraprocedural perforation (*n* = 2), delayed bleeding (*n* = 4), and thrombophlebitis (*n* = 1). Intraprocedural perforations were endoscopically clipped shut, administered intravenous antibiotics, and the ESDs were completed.

Of the 24 patients with non‐curative resection, 18 (16.1%) were referred for surgery, with the others declining referral due to frailty or patient preference. Of the 18 referred for surgery, four (3.6%) patients underwent gastric surgery post ESD (including one resection which was abandoned due to submucosal scarring). No patients (0%) had positive lymph nodes. One patient with an eCuraC2 lesion was found to have residual adenocarcinoma in the surgically resected stomach; however, margins were clear and there was no lymphovascular invasion. There was no residual disease or involved LN in the resection specimens of the remaining three patients that underwent surgery. Of the 14 patients who were referred but did not undergo surgery, the median age was 72.5 years [9.7 years], with a mortality rate of 35.3% (*n* = 6) over a median duration of 7.5 months, reflecting the highly comorbid nature of this group. During continuous follow‐up of this cohort, none (0%) of the surviving patients have developed recurrence or metastatic disease.

Ten (8.9%) patients developed metachronous lesions. Seven patients (six adenocarcinoma and one HGD) had repeat curative ESD; one patient had a repeat ESD which was abandoned, and the patient proceeded to surgery; one patient was diagnosed with a new LGD; one patient refused further treatment and returned to their country of origin.

All surviving patients (100%) were reviewed within 3 months of the end of the study period, with the median duration of follow‐up (surveillance gastroscopy, CT, or clinic appointment) being 158 weeks, and the maximum duration being 579 weeks (11.1 years). There were 16 deaths during the course of follow‐up, none of which were related to gastric cancer (heart failure 30.8%, kidney disease 7.7%, and cholangiocarcinoma 7.7%).

### Risk stratification scores

The median eCura score was 0, with 86 (76.8%) in the eCura A, 2 (1.8%) in eCura B, 2 (1.8%) in eCura C1, and 22 (19.6%) in the eCura C2 categories (Table 2). Descriptions of non‐curative lesions are seen in Tables 3 and 4. There were no differences in categorization between eCura or ESGE. One additional patient was changed from eCura C2 to W‐eCura C1 due to a depth of invasion of 600 nm, resulting in a down‐grade. This would not have made any influence on post‐resection strategy.

**Table 3:** Comparison of outcomes by curative status

Variables	Curative (*n* = 88)	Non‐curative (*n* = 24)	*P* value
Age, median, [IQR]	76 years [12 years]	72 years [14 years]	0.88
Size, median, [IQR]	20 m [8.9 mm]	33 mm [27.2 mm]	<0.001*
Previous/existing H pylori	58 (22.7%)	2 (9.5%)	0.23
Autoimmune Gastritis	20 (23.5%)	9 (36%)	0.21
Previous gastric surgery	3 (3.6%)	2 (8.3%)	0.31
Location			0.41
Antrum	35 (39.3%)	6 (26.1%)	
Body	29 (32.6%)	11 (47.8%)	
Incisura	14 (15.7%)	2 (8.7%)	
Cardia	9 (10.1%)	4 (17.4%)	
Fundus	2 (2.2%)	–	
Paris classification			0.64
Is	13 (16.7)	2 (10%)	
IIa	50 (64.1%)	16 (80%)	
IIb	11 (14.1%)	1 (5%)	
IIc	4 (5.1%)	1 (5%)	
Stage			<0.001*
pT1a	53 (59.6%)	10 (45.5%)	
pT1b	3 (3.4%)	11 (50%)	
pT2	–	1 (4.5%)	
IEN	33 (37.1%)	–	
Differentiation			<0.001*
Well/moderate	52 (96.3%)	12 (60%)	
Poor	2 (3.7%)	8 (40%)	
Duration	240 min [124 min]	120 min [56.2 min]	<0.001*
Complications	12 (13.5%)	2 (9.1%)	0.73
Recurrence	0%	0%	—
Metachronous Lesions	8 (9.4%)	3 (13.6%)	0.85
Mortality	8 (10.8%)	7 (31.8%)	0.02*
Disease related	0%	0%	—
Time to death	140 [123]	151 [113]	0.73
Duration of follow‐up	202 [125 weeks]	131 [71 weeks]	0.006*

Table comparing outcomes between groups defined as curative *versus* non‐curative.

IEN, intraepithelial neoplasia.

**Table 4:** Characteristics of individual non‐curative lesions

Individual lesions	En bloc	R0	Size	Differentiation	Ulcer	LVI	Submucosal Invasion
eCura C1							
#1	Yes	No	15 mm	Poor	No	No	No
#2	No	No	130 mm	Well	No	No	No
eCura C2							
#1	Yes	Yes	50 mm	Poor	No	No	No
#2	No	No	25 mm	Poor	Yes	No	1400 μm
#3	Yes	Yes	35 mm	Poor	No	No	No
#4	Yes	Yes	35 mm	Well	No	Yes	1300 μm
#5	No	No	50 mm	Poor	No	No	T2
#6	Yes	Yes	12 mm	Well	No	Yes	100 μm
#7	Yes	Yes	10 mm	Well	Yes	Yes	No
#8	Yes	Yes	70 mm	Well	No	No	100 μm
#9	Yes	Yes	30 mm	Well	No	Yes	500 μm
#10	Yes	Yes	17 mm	Well	No	Yes	No
#11	Yes	No	33 mm	Moderate	No	No	600 μm
#12	Yes	Yes	70 mm	Poor	No	No	No
#13	Yes	Yes	50 mm	Poor	No	Yes	No
#14	Yes	Yes	80 mm	Poor	Yes	No	No
#15	Yes	Yes	15 mm	Yes	No	Yes	1200 μm
#16	Yes	No	20 mm	Well	No	No	1700 μm
#17	Yes	Yes	25 mm	Poor	No	Yes	300 μm
#18	Yes	Yes	25 mm	Poor	No	No	No
#19	Yes	Yes	30 mm	Poor	No	No	No
#20	Yes	No	30 mm	Well	No	Yes	1900 μm
#21	Yes	Yes	60 mm	Well	Yes	No	No
#22	No	No	46 mm	Moderate	No	Yes	500 μm

Table describing characteristics of individual ‘non‐curative’ lesions.

### Comparison between curative and non‐curative lesions

Comparison of the characteristics of curative and non‐curative lesions can be seen in Table 3. Univariate and multivariate analysis of the factors associated with resection, complications, metachronous lesions, and mortality are seen in Table 5. Multivariate analysis demonstrated that longer duration was associated with non‐curative resection.

**Table 5:** Univariate and multivariate analysis of associations between outcomes and lesion characteristics

Factors	OR (95% CI)	*P* value
En bloc resection		
Size	1.06 (1.02–1.11)	0.009*
R1 resection		
Size	1.05 (1.02–1.09)	0.003*
Duration	1.02 (1–1.04)	0.018*
Previous gastric surgery	6.89 (1.01–46.78)	0.048*
Multivariate analysis		
Size	1.06 (0.93–1.2)	0.39
Duration	1.01 (0.98–1.04)	0.5
Previous gastric surgery	22.6 (0.37–1376.5)	0.14
Non‐curative resection (eCura)		
Size	1.09 (1.04–1.13)	<0.001*
Duration	1.04 (1.01–1.07)	0.004*
Differentiation	4.92 (2.14–11.29)	<0.001*
Multivariate analysis		
Duration	1.04 (1.003–1.07)	0.03*
Size	1.06 (0.95–1.17)	0.31
Differentiation	15 808 (0 – Inf)	0.99
Complications		
Autoimmune gastritis	4.69 (1.47–15.1)	0.009*
Metachronous lesions		
*H. pylori*	5.07 (1.29–19.87)	0.02*

* = *p* < 0.05.

### Comparison with Western data

**Figure 4:**
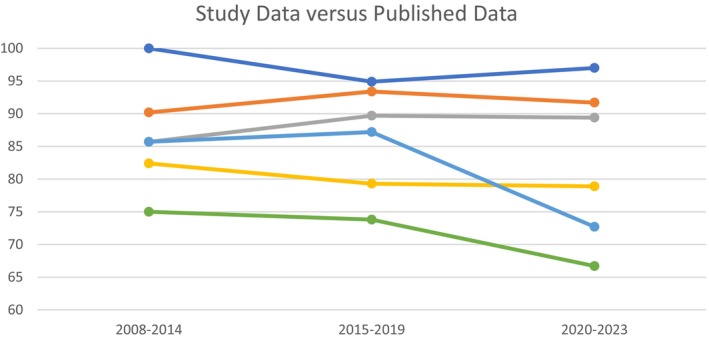
Comparative data between current study and existing published data. Comparison between rates of en bloc, complete and curative resection between the current study and published series, when compared over time. RPH = Royal Perth Hospital, R0 = complete resection with negative margins. RPH en bloc, 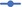; RPH R0, 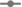; RPH Curative, 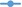; Pooled en bloc, 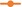; Pooled R0, 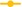; Pooled Curative, 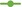.

Review of available published data on Western outcomes from gastric ESD revealed a total of 35 relevant articles from 2008 to 2023^10^, ^16^, ^17^, ^18^, ^19^, ^20^, ^21^, ^22^, ^23^, ^24^, ^25^, ^26^, ^27^, ^28^, ^29^, ^30^, ^31^, ^32^, ^33^, ^34^, ^35^, ^36^, ^37^, ^38^, ^39^, ^40^, ^41^, ^42^, ^43^, ^44^, ^45^, ^46^, ^47^ (Table 6). There was a total of 3589 lesions, with an analysis of outcomes over the entire period returning a mean en bloc resection rate of 94.8%, R0 resection rate of 84.2%, and curative resection of 69.8%. When divided by period, there was an increase in the number of lesions studied between 2008 and 14 (209), 2015 and 19 (1001), and 2020 and 23 (2379). When evaluating en bloc rates between periods, there were no differences in mean rates (90.2%, 93.4% and 91.7% respectively, *P* = 0.718). Similarly, there were no differences in R0 resection rates (82.4%, 79.3% and 78.9% respectively, *P* = 0.88) or curative resection rates (75%, 73.8% and 66.7% respectively, *P* = 0.95). By comparison, our data demonstrated en bloc rates of 100%, 94.9% and 97% (*P* = 0.75) across the same time period (Fig. 4). R0 rates were 85.7%, 89.7% and 89.4% (*P* = 0.95) and curative resection rates were 85.7%, 87.2% and 72.7% (*P* = 0.19).

**Table 6:** Literature review of outcomes of gastric ESD

Author	Year	Lesions (*n*)	En Bloc (%)	R0 (%)	Curative (%)
Cardoso	2008	15	80	60	75
Catalano	2009	12	91.7	91.7	—
Coda	2010	7	85.7	85.7	—
Hulagu	2011	24	91.7	91.7	—
Schumaker	2012	30	90	60	—
Baldaque‐Silva	2013	17	100	100	—
Repici	2013	42	100	92.9	—
Chavez	2013	62	82.3	77.4	—
Jaques	2015	6	100	83.3	—
Donoso	2015	16	100	87.5	87.5
Emura	2015	54	98.1	92.6	—
Najmeh	2016	30	100	86.7	—
Karpinska	2016	58	96.6	81	70.7
Sooltangos	2017	25	60	32	—
Libanio	2017	194	95.4	93.8	—
Probst	2017	191	92.1	75.9	63.9
Chirinos Vega	2018	13	84.6	84.6	—
Mendonca	2018	51	92.2	72.5	71.1
Petruzzielo	2018	70	97.1	62.9	—
Mocker	2019	26	100	80.8	76
Costa	2019	114	96.5	86.8	—
Libanio	2019	153	94.8	90.2	—
Chen	2020	42	81	76.2	—
Maselli	2020	502	84.5	82.3	—
Canete Ruiz	2020	35	85.7	80	77.1
Manta	2020	299	97.7	89	72.5
Kim	2020	46	89.1	23.9	18.9
Dragonov	2021	101	98	82.2	—
Fernandez‐ Esparrach	2021	230	91.3	75.2	—
Doumbe‐Mandengue	2021	19	89.5	78.9	63.2
Ngamruengphong	2021	311	92.3	83	58.7
Fleischmann	2021	236	92.4	80.5	—
Mejia	2021	102	98	93.1	87
Da Silva Costa	2022	41	97.6	97.6	80.5
Bhandari	2023	415	94.7	83.4	75.8

* = *p* < 0.05. Outcomes of published data of outcomes of gastric ESD by time, number of lesions, en bloc, complete resection and curative resection.

## Discussion

This study demonstrates that ESD for gastric neoplasia is associated with high rates of technical and clinical success in a multicultural tertiary referral center in Australia.

Historically, East Asian outcomes have been superior to those seen in Western centers, as demonstrated in a systematic review and meta‐analysis by Daoud et al.^7^ However, as Western experience continues to develop, so too have outcomes improved. Recent studies from Europe and North America have demonstrated comparable rates of en bloc resection, R0 resection and adverse events.^9^, ^36^ Our study reports rates of en bloc resection (96.4%), complete or R0 resection (89.3%), and curative resection (78.6%) comparable with Asian and international series, with a slight decline in curative resection rates in the latter tertile, thought to be due to increasing complexity of cases accepted for resection.^48^

Previous studies has reported the rate of non‐curative ESD for EGC at approximately 20%. This is associated with an increased risk of LNM or parietal recurrence.^3^, ^11^ Regression analysis of our cohort demonstrated that on multivariate analysis, duration of procedure was the only factor associated with non‐curative resection. This may reflect a more difficult procedure or potentially larger lesions, although this was not borne out on analysis. None of location, Paris classification, age, ethnicity, H pylori, or autoimmune gastritis status incurred greater risk of non‐curative resection, which is of interest and warrants further exploration.

The eCura system was validated in 2017 and is incorporated into JGES guidelines to direct post‐resection management.^12^ Since its inception, our center has used eCura to define curativeness and guide management. This study demonstrates that among patients with curative resection as defined by eCura A or B, there were no cases of recurrence and 11 metachronous lesions, with a median duration of follow‐up of 3.9 years. In high‐risk lesions (eCura C1/2) who underwent surgery, there was one case of residual tumor, no LNM, no recurrence and three metachronous lesions. In our cohort of high‐ risk lesions who did not undergo surgery, we followed these patients up endoscopically and with CT imaging. In this cohort, seven (31.8%) died from non‐disease related comorbidities. We would advise against surgery for non‐curative lesions in the very elderly and those with significant comorbidities.

As yet, there is no validated system comparable to eCura for use in the Western setting. However, a recent study by Morais et al. has proposed a modified version following evaluation of over 300 non‐curative ESD across European and Australian centers.^13^ On analysis of risk factors for LNM, submucosal invasion ≥1 mm was found to correlate better than >0.5 mm. Reflecting this, the W‐eCura system was created. The W‐eCura score was associated with an AUC‐ROC for LNM (0.916, 95% CI 0.870–0.961), significantly better than the original in this cohort.^13^

We aimed to assess the applicability of eCura, W‐eCura, and also ESGE to determine if there were any differences between the scoring systems. We determined that one patient was downgraded from eCura C2 to W‐eCura C1 on the basis of submucosal invasion of 600 nm. They were referred to surgery but declined due to frailty and age. While this would not result in a practical difference in strategy, evidence does suggest that the risk of metastasis in C1 lesions is low. JGES suggest that repeat endotherapy or follow‐up are reasonable strategies. There were no other relevant differences between the systems. This is reassuring and suggests that familiarity with a single tool may be equally beneficial in guiding post‐resection management but is a space which requires further larger studies to evaluate potential subtle differences that may influence outcomes for patients in Western centers.

The incidence of metachronous lesions requires regular follow‐up of patients post ESD. This phenomenon is likely as a result of a field defect, such as the ongoing risk of gastric cancer from autoimmune gastritis. Our rate of metachronous lesions was 9.9% among all cases, which is in line with international series such as that by Abe et al. who demonstrated a 5‐year incidence of 9.5%.^49^ It also highlights the importance of surveillance gastroscopy post ESD to detect such lesions, even where resections have been deemed curative.

Initial rates of adverse events during Western gastric ESD were significantly higher than corresponding Japanese series.^50^ This has been considered as one the reasons influencing the more gradual adoption of ESD outside of East Asia. Our study is in line with more recent data supporting the safety of ESD. Our rates of adverse events are 5.8% overall, with the majority of these complications relating to delayed bleeding (3.4%). These figures are comparable with other Western data and once again support the safety of ESD for EGC outside of Japan.^51^

Our study benefits from being a single‐center study with a large number of EGC, thereby limiting heterogeneity and adding to the real‐world data on ESD for such lesions outside of Japan. As such, the cohort is multicultural with a mix of ethnicities, with Caucasians making up the majority. We also benefit from reporting on over 13 years of data, with regular follow‐up in all patients. We aimed to compare our data with other Western series through a comparative literature review. This demonstrated favorable comparability. The volume of cases and lesions has steadily increased over time, reflecting Western adoption and experience. Interestingly despite this, neither our series nor the pooled international data show a significant change in outcomes over time. While acknowledging this review does not amount to a formal meta‐analysis, it could be seen to illustrate a combination of increased caution during early procedures, bias, or perhaps that target outcomes may be more user‐dependent rather than reliant on advancements in technology.

Whilst this was a retrospective, single‐center observational study, we sought to limit bias by establishing a comprehensive, standardized template for documentation of procedural and patient data, and a standard histopathological reporting template in line with WHO guidance. We compared several different risk stratification tools including eCura, W‐eCura, and ESGE guidelines and found that outcomes correlated well with risk of recurrence or LNM. We sought to compare our data with other series to demonstrate comparability. Our numbers of surgical resections were low, which limits conclusions relating to non‐curative resections; however, close surveillance with CT and gastroscopy failed to detect any recurrence or LNM to date.

In conclusion, this study demonstrates that high rates of safe, clinically, and technically successful ESD for EGC can be achieved in the Western setting. Numbers of lesions resected have steadily increased over time, with outcomes comparable across time periods. Risk stratification tool such as eCura can be used to practically and successfully direct management in patients post ESD. Further long‐term prospective research utilizing evaluating the validity of risk stratification tools in a Western cohort will be useful.
